# Heart Rate Response During Treadmill Exercise Test in Children and Adolescents With Congenital Heart Disease

**DOI:** 10.3389/fped.2019.00065

**Published:** 2019-03-12

**Authors:** Fabian von Scheidt, Stephanie Meier, Johannes Krämer, Anita Apitz, Jannos Siaplaouras, Peter Bride, Michael Kaestner, Christian Apitz

**Affiliations:** Division of Pediatric Cardiology, Children's Hospital, University of Ulm, Ulm, Germany

**Keywords:** heart rate response, chronotropic incompetence, heart rate recovery, cardiopulmonary exercise testing, congenital heart disease

## Abstract

**Background:** Impaired exercise capacity is a common feature of congenital heart disease (CHD). In adults with CHD, it has been shown that impaired heart rate response during exercise may contribute to exercise limitation. Systematic data in children and adolescents on this topic is limited. We therefore purposed to assess heart rate response during treadmill exercise testing in children and adolescents with CHD compared to healthy controls.

**Methods:** One hundred and sixty three children and adolescents (103 with CHD, median age 15 years and 60 age-matched controls) performed cardiopulmonary exercise testing and were included in this study. Beyond peak oxygen consumption, increase in heart rate from resting level to peak exercise (heart rate reserve) and decrease of heart rate after peak exercise (heart rate recovery) were measured. Chronotropic index was defined as percentage of age predicted maximal heart rate reserve. According to data from adults on bicycle exercise, chronotropic incompetence was assumed for chronotropic index below 0.8.

**Results:** While resting heart rate was similar between both groups, peak heart rate, heart rate reserve as well as chronotropic index were lower in the CHD group than in controls. Chronotropic index was lowest in patients with single ventricle hemodynamics and correlated with peak oxygen consumption. Heart rate recovery was impaired in the CHD group 1 and 2 min after peak exercise compared to controls and correlated with peak oxygen consumption. Chronotropic index below 0.8 was a relatively frequent finding even in the control group suggesting that the threshold of 0.8 appears inadequate for the identification of chronotropic incompetence using treadmill exercise testing in children. After normalizing to the 2.5th chronotropic index percentile of the control group we obtained a chronotropic incompetence threshold of 0.69.

**Conclusion:** As an adjunct to measurement of peak oxygen consumption, heart rate response to exercise appears to be a physiologically important diagnostic parameter in children and adolescents with CHD. However, interpretation of heart rate response needs to consider specific age characteristics and the mode of exercise test. Our data may help future studies on chronotropic incompetence using treadmill ergometer protocols in children and adolescents.

## Introduction

Congenital heart disease (CHD) are among the most common congenital defects. Every year ~1.5 million children worldwide are born with CHD (1). Progress in medical care has led to an improved survival of patients after surgical repair of CHD. Nowadays ~90 percent of CHD patients have the prospect to reach adulthood (2, 3).

However, many CHD patients suffer from impaired exercise capacity. The assessment of peak oxygen consumption is the gold standard for the assessment of exercise tolerance (4) and has been widely used for evaluation of exercise capacity in individuals with CHD (5). In adults with CHD, it has been shown that impaired heart rate response during bicycle exercise may contribute to exercise limitation (6). Heart rate response during exercise is a complex composition of chronotropic reserve during exercise and heart rate recovery after cessation of exercise. Interaction of the sympathetic and parasympathetic nervous system plays an important role in the modulation of heart rate during exercise (7). In CHD patients this may be affected for different reasons including ischemia and/or denervation resulting from surgical procedure or in case of cyanotic CHD from chronic hypoxemia (8).

Systematic data in children and adolescents on this topic are limited and might differ from adult data due to different reasons: Firstly, differences in cardiorespiratory response to exercise between adults and children exist. In children a higher total peripheral resistance is seen. Smaller muscle mass and smaller heart size in children result in lower venous return and contribute to a lower stroke volume making the increase in heart rate the most important compensatory mechanism to increase cardiac output (9, 10). Therefore, heart rate response during exercise may play a more crucial role in the assessment of exercise limitation in children and adolescents than in adults.

Secondly, the mode of exercise testing may play a relevant role, as most frequently exercise testing in children is performed using a treadmill rather than a bicycle as in adults (11). Hereby upright body posture could result in higher resting heart rates, a phenomenon known from tilt table test, which may have impact on the interpretation of chronotropic response (12). We therefore pursued to assess heart rate response during treadmill exercise test and its impact on exercise performance in children and adolescents with CHD compared to healthy controls.

## Methods

### Study Population

We retrospectively analyzed all evaluable cardiopulmonary exercise tests (CPET) performed in an outpatient clinic setting at our referral center between January 2015 and December 2016. Patients were referred for exercise testing as part of routine clinical follow-up protocols used for patients with CHD at our institution. Informed consent was obtained from all patients undergoing exercise testing. Almost all patients underwent only one test during the period; for those who underwent two tests we addressed only the first test. Patients treated with betablockers or pacemakers were excluded. A main diagnosis was determined for every patient from hospital records.

One hundred sixty-three examinations of children and young adults were eligible for analysis, including 103 examinations in CHD patients with varying underlying heart disease.

Sixty age-matched normal pediatric patients served as controls who had no cardiac defects or family history of cardiac disease or they were patients who were evaluated in the cardiology clinic for heart murmurs, chest pain, or syncope with normal structure hearts.

The study protocol conforms to the ethical guidelines of the 1975 Declaration of Helsinki and was approved by the local ethics committee.

### Cardiopulmonary Exercise Testing

CPET was performed on an h/p/cosmos mercury med (Nussdorf-Traunstein, Germany) treadmill according to a modified Bruce protocol as described previously by Dubowy et al. (11). All subjects were encouraged to exercise until exhaustion regardless of the maximal heart rate achieved. Ventilation, oxygen uptake, and carbon dioxide production were measured continuously. Heart rate was assessed with 12-lead electrocardiography. Resting heart rate was measured after at least 2 min in an upright standing position and peak heart rate was defined as the maximal heart rate achieved during exercise. All patients with a respiratory exchange ratio (RER) at peak exercise ≥1.01 while reaching the second ventilatory threshold or a RER ≥1.04 as previously described by other study groups (13, 14) were considered to have performed a maximal CPET.

### Heart Rate Reserve, Chronotropic Index, and Chronotropic Incompetence

Heart rate reserve was calculated as the difference between peak and resting heart rate. Chronotropic index was calculated by a formula [(peak heart rate–resting heart rate)/(220–age–resting heart rate)] derived from the chronotropic metabolic relationship concept introduced by Wilkoff and Miller (15). Thus, facilitating comparability of a normal chronotropic response irrespectively of age, resting heart rate, and functional state. In a group of 410 healthy adults Wilkoff and Miller reported 95% limits of normality for chronotropic index to be 0.8–1.3. Based on this finding, chronotropic incompetence is usually defined as failure to achieve a chronotropic index of 0.8 or higher (i.e., falling below 97.5 percent of healthy adults).

### Heart Rate Recovery

Heart rate recovery (HRR) was recorded as decrease of heart rate 1, 2, and 3 min after cessation of peak exercise. Relative HRR was calculated dividing the heart rate after 1, 2, and 3 min by the heart rate at peak exercise.

### Statistical Analysis

All values are given as mean ± standard deviation unless otherwise stated. Comparison between CHD- and non-CHD (sub)-groups was made using Mann-Whitney-*U*-test. For correlation analysis Spearman-Rho test was applied. Statistical analysis was performed using SPSS Statistics for Windows, version 24.0 (IBM Corp. Released 2016, Armonk, NY). *P*-values ≤ 0.05 were considered statistically significant.

## Results

### Study Group Characteristics

Subgroup apportionment for the CHD cohort is shown in Table 1. Control and CHD group showed similar gender distribution. Age, weight, height, and body mass index did not differ substantially. Main demographic characteristics and CPET findings are listed in Tables 2A,B.

**Table 1:** Study group characteristics.

**Controls *n* = 60 (47% female)**	**Congenital heart disease (CHD) *n* = 103 (46% female)**	
	Fontan circulation	*n* = 12 (42% Female)
	Pulmonary arterial hypertension (PAH)	*n* = 12 (58% Female)
	Complex anatomy, biventricular corrected	*n* = 18 (33% Female)
	Tetralogy of Fallot	*n* = 21 (67% Female)
	D-Transposition of the great arteries (TGA)	*n* = 12 (50% Female)
	Septal defects	*n* = 13 (46% Female)
	Valvular defects	*n* = 15 (20% Female)

**Table 2:** Demographic and CPET data.

	**Controls**	**CHD**	
**A DEMOGRAPHIC DATA**			
Age [years]	13.0 ± 3.8	14.8 ± 4.8	
Weight [kg]	50.1 ± 15.7	54.4 ± 20.6	
Height [cm]	157.2 ± 16.3	159.6 ± 17.9	
BMI [kg/m^2^]	19.8 ± 3.4	20.5 ± 4.5	
	**Controls**	**CHD**	***p***
**B CPET DATA**			
Loading time [min]	11:53 ± 1:35	10:39 ± 2:02	<0.001
Distance [m]	905.9 ± 217.6	803.5 ± 214.0	<0.01
Speed [km/h]	6.2 ± 0.5	5.7 ± 0.7	<0.001
METS	14.8 ± 1.8	13.1 ± 2.7	<0.001
Peak VO_2_ [l/min]	2.1 ± 0.73	1.8 ± 0.75	<0.05
Indexed peak VO_2_ [ml/min/kg]	41.7 ± 7.0	34.3 ± 7.7	<0.001
V'/E [l/min]	63.5 ± 21.7	62.0 ± 22.9	n.s.
V'E/V'O_2_	28.8 ± 3.6	32.3 ± 6.0	<0.001
V'E/V'CO_2_	27.6 ± 2.8	30.6 ± 5.4	<0.01
RER	1.04 ± 0.08	1.06 ± 0.08	n.s.
VT	1.49 ± 0.50	1.50 ± 0.67	n.s.
RR [min^−1^]	43.2 ± 7.3	43.6 ± 10.1	n.s.

*CPET, Cardiopulmonary exercise testing; METS, Metabolic equivalent; Peak VO_2_, Peak oxygen consumption; V'/E, minute ventilation; V'E/V'O_2_, Ventilatory equivalent of oxygen; V'E/V'CO_2_, Ventilatory equivalent of carbon dioxide; RER, Respiratory exchange ratio; VT, Ventilatory threshold; RR, Maximum respiratory rate. All parameters given at peak VO_2_*.

### Chronotropic Index and Chronotropic Incompetence

**Figure 1:**
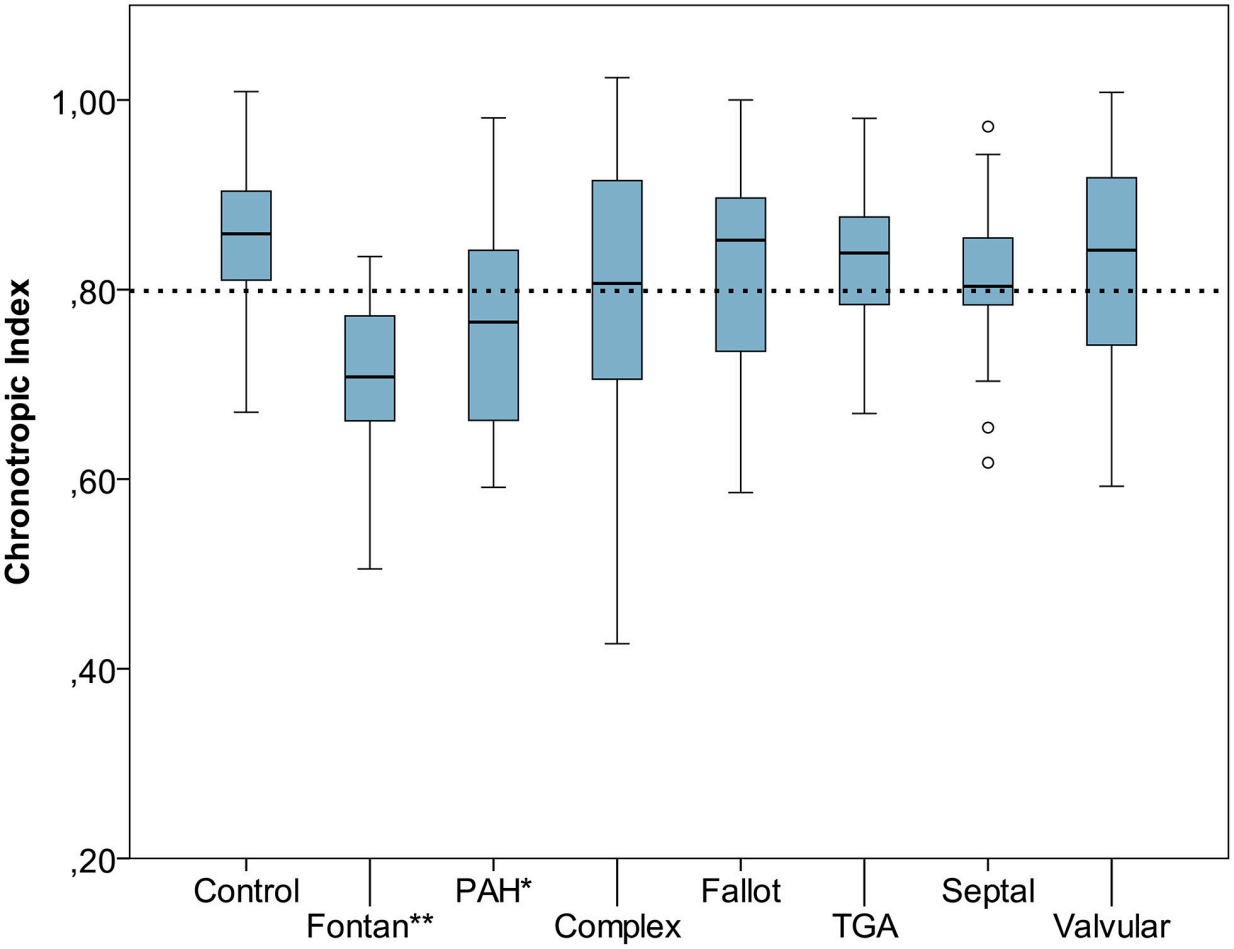
Chronotropic index [Chronotropic index = (peak heart rate–resting heart rate)/(220–age–resting heart rate)] of control group vs. congenital heart disease (CHD) subgroups. Dashed line marks chronotropic incompetence threshold of 0.8. ^*^p < 0.05, ^**^p < 0.001. Boxplot bottom indicates 25th, top of the box 75th percentile. Whiskers extend to 1.5 times of box height or to minimum/maximum value. Outliers are shown as points.

**Figure 2:**
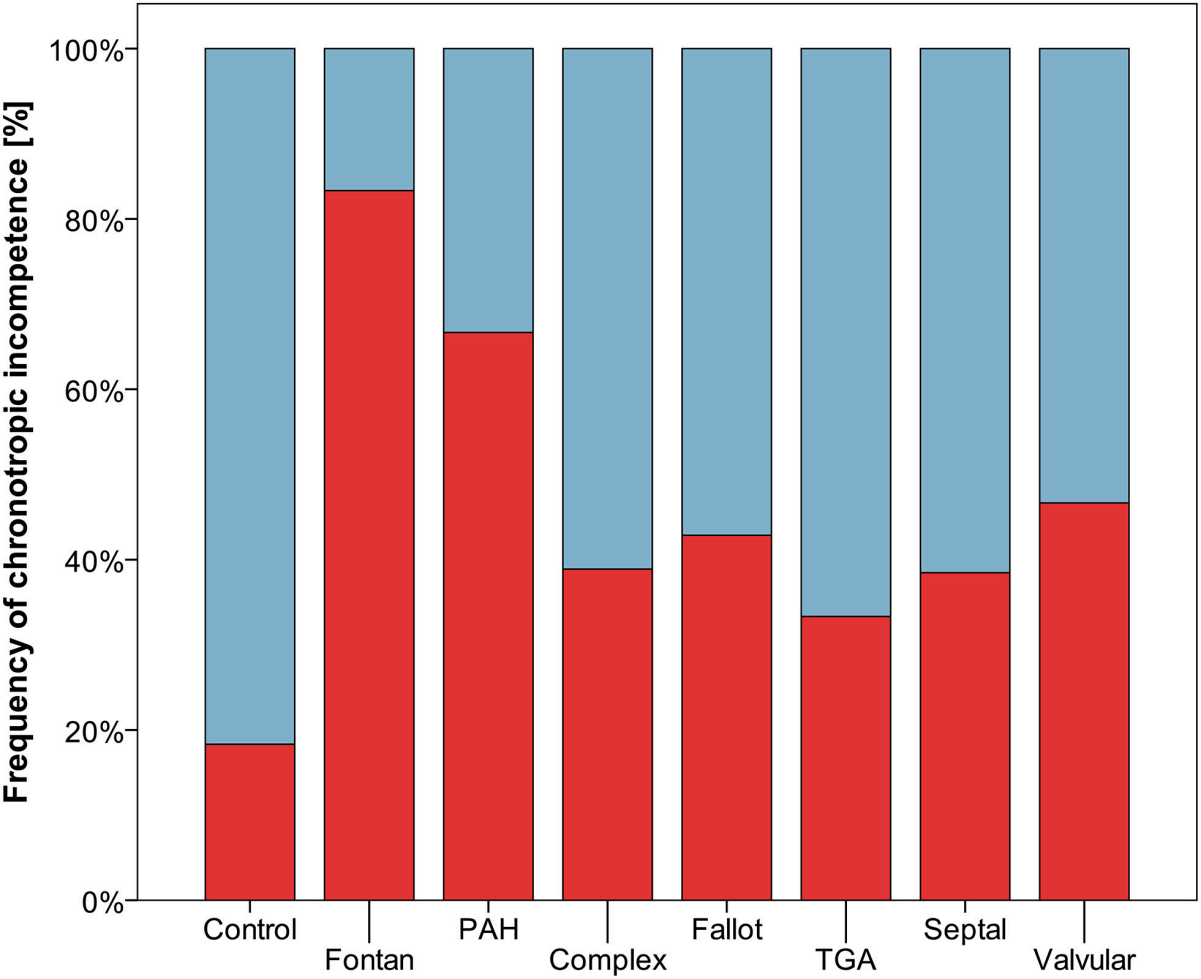
Frequency of chronotropic incompetence (threshold defined as chronotropic index < 0.8) marked red.

**Figure 3:**
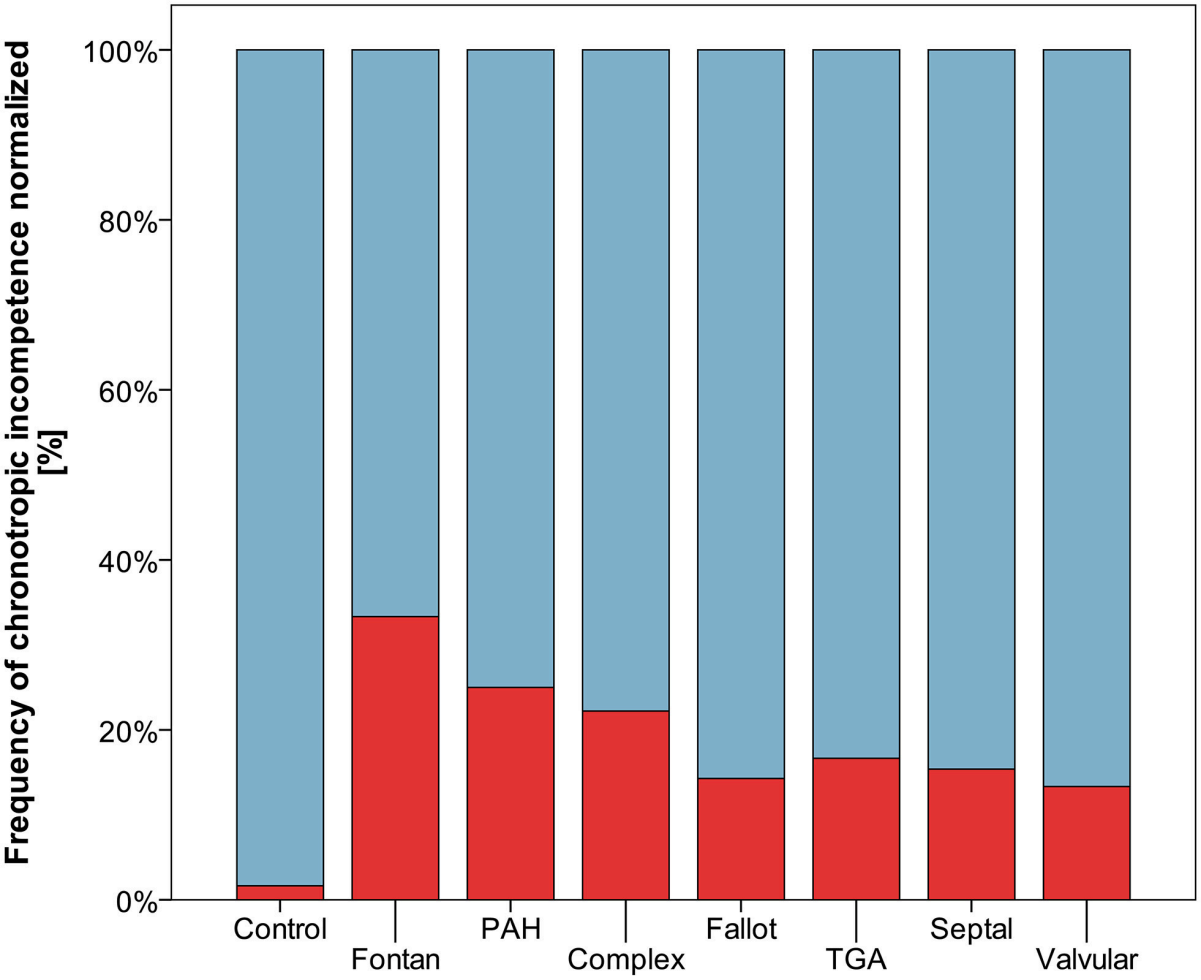
Frequency of chronotropic incompetence normalized to healthy controls (threshold defined as chronotropic index < 0.69 corresponding to the 2.5th percentile of chronotropic index in controls) marked red.

Resting heart rate was similar between study groups. Peak heart rate, heart rate reserve as well as chronotropic index were lower in the CHD group than in controls, details shown in Table 3. Chronotropic index was lowest in patients with Fontan circulation as shown in Figure 1. Chronotropic index was related to peak oxygen consumption (Spearman-rho correlation coefficient 0.25). Frequency of chronotropic incompetence in the study subgroups is displayed in Figure 2. Defining a chronotropic index of 0.8 as chronotropic incompetence threshold revealed a relatively high rate of chronotropic incompetence even in the control group (Figure 2), most likely due to an overestimation of the maximal heart rate using the (220-age) formula. Therefore, Figure 3 is normalized to a chronotropic incompetence threshold defined as the 2.5th percentile of chronotropic index in the control group.

**Table 3:** Heart rate characteristics during CPET.

		**Control**	**CHD**	***p***
Resting HR [bpm]		85.0 ± 14.6	83.3 ± 13.3	0.81
Peak exercise HR [bpm]		189.0 ± 8.3	180.0 ± 13.3	<0.001
HR reserve [bpm]		104.0 ± 13.6	96.2 ± 18.0	0.004
Chronotropic index		0.85 ± 0.07	0.79 ± 0.11	0.001
1 min HRR				
Absolute [bpm]		−37.8 ± 15.0	−30.2 ± 12.4	0.002
Relative		0.80 ± 0.08	0.83 ± 0.07	0.01
2 min HRR				
Absolute [bpm]		−57.1 ± 12.8	−50.3 ± 14.5	0.004
Relative		0.70 ± 0.07	0.72 ± 0.08	0.05
3 min HRR				
Absolute [bpm]		−64.0 ± 13.2	−59.1 ± 13.7	0.10
Relative		0.66 ± 0.07	0.67 ± 0.07	0.58

*Heart rate characteristics during cardiopulmonary exercise testing. Values as mean ± standard deviation. CHD, Congenital heart disease; HR, heart rate; HRR, heart rate recovery (HR decrease from peak exercise); bpm, beats per minute*.

### Heart Rate Recovery

**Figure 4:**
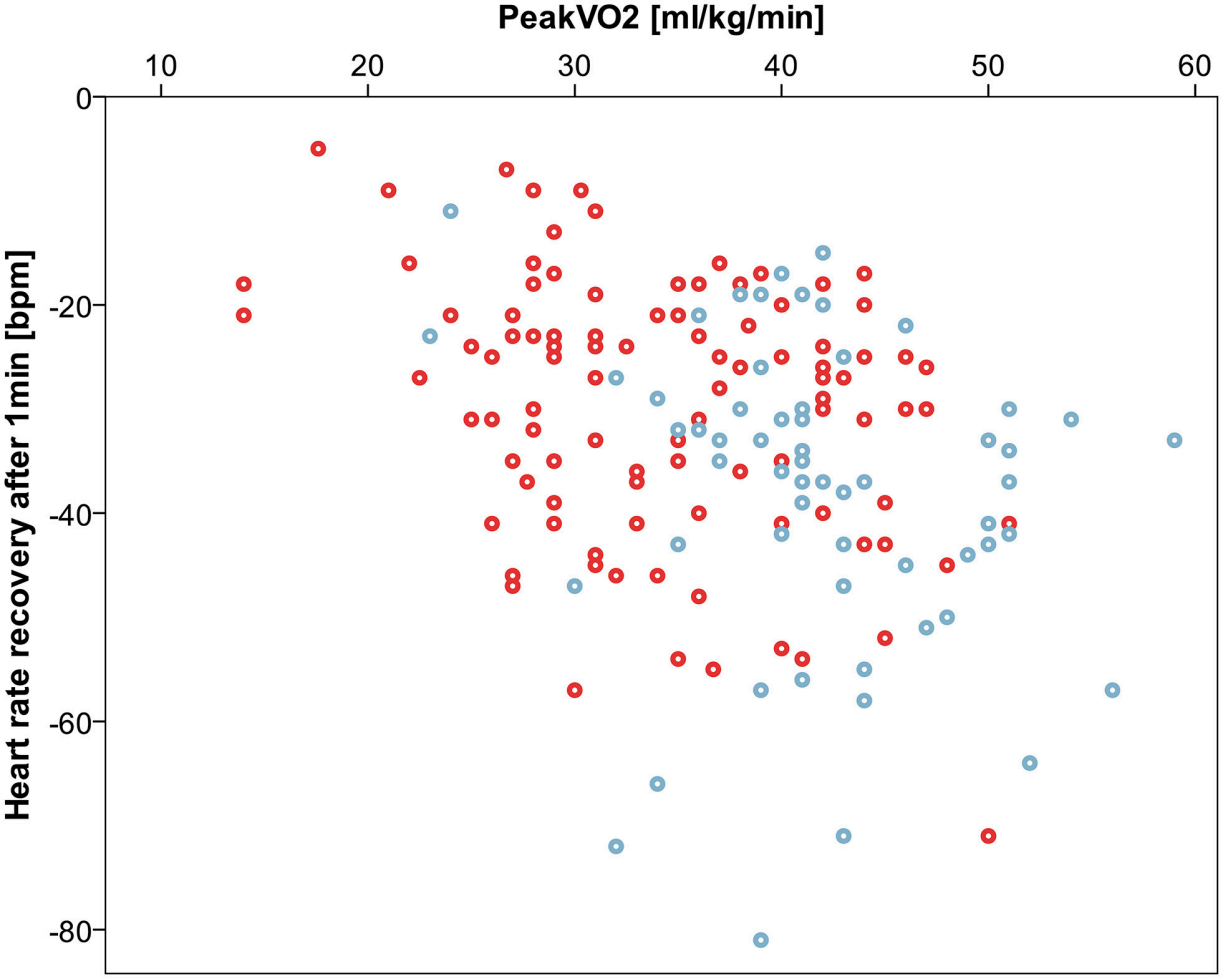
Correlation between 1 min heart rate recovery (HRR) and peak oxygen consumption (peak VO_2_). Spearmans-rho correlation coefficient −0.35. Controls displayed blue, congenital heart disease red.

HRR measured as heart rate decrease from peak exercise heart rate within a defined interval was impaired in CHD patients compared to controls after 1 and 2 min and converged 3 min after peak exercise level. Concordantly, relative HRR was higher in the CHD group after 1 and 2 min confirming a deferred heart rate decrease. Details are listed in Table 3. Correlation of HRR and peak oxygen consumption was noteworthy with Spearmans-rho correlation coefficients of −0.35 after 1 min, −0.43 after 2 min, −0.37 after 3 min. Figure 4 shows a scatterplot of 1 min HRR and peak oxygen consumption. HRR was most impaired in the PAH subgroup compared to healthy controls with a HRR of −22.2 ± 13.7 bpm (relative HRR 0.87) after 1 min, −36.7 ± 15.2 bpm (relative HRR 0.79) after 2 min and −44.1 ± 15.5 bpm (relative HRR 0.75) after 3 min, all *p*-values < 0.01 compared to healthy controls.

## Discussion

To the best of our knowledge, this study is the first to assess heart rate response during treadmill exercise in children and adolescents with CHD. Our results show that as an adjunct to the measurement of peak oxygen consumption, heart rate response during exercise appears to be a physiologically important diagnostic parameter in children and adolescents with CHD.

Our data may help to interpret future studies on chronotropic incompetence using treadmill ergometer protocols in children and adolescents.

### Chronotropic Incompetence

The limited increase in heart rate during exercise (chronotropic incompetence) is known as a predictor of mortality in adult patients with coronary artery disease and in healthy adult population (16–18). Diller and colleagues detected impaired chronotropic response during exercise also in an adult CHD cohort, even with a prevalence of up to 62% (6).

Data regarding chronotropic response during exercise in children and adolescents is scarce. Our results highlight the importance to consider age specific characteristics and the mode of exercise test when interpreting heart rate response in children and adolescents. This is particularly apparent for the calculation of chronotropic index. The used formula to calculate chronotropic index (CI) is intended to normalize chronotropic response independently of age but it was developed for an adult cohort. Threshold definition for CI is variable throughout literature (19). In the original publication Wilkoff and Miller reported 95% limits of normality for chronotropic index to be 0.8–1.3 in a group of 410 healthy adults (15). Hereby CI had been defined as failure to achieve a chronotropic index of 0.8, i.e., falling below the 2.5th percentile of healthy adults, while 2.28th percentile represents −2 standard deviations, however rounding up to 2.5th percentile has been done before when reporting age related normal ranges (13). Whether the CI threshold of 0.8 is applicable in children and young adults has not been demonstrated so far and raises doubt. Using the (220-age) formula it is likely to overestimate the maximum heart rate of young subjects (20, 21). In addition, the above named reference dataset was assessed using a bicycle ergometer. The use of a treadmill ergometer as in our study might theoretically result in a higher resting heart rate level by recording the heart rate in an upright standing position and not in a sitting position. The resting heart rate is included in the calculation of the chronotropic index and thus a higher resting heart rate results in lower values for chronotropic index. This might explain the relatively high rate of CI in our control group applying a CI threshold of 0.8.

To cope with this bias, we adopted the above mentioned concept of Wilkoff and Miller, and normalized our study cohort to the 2.5th chronotropic index percentile of the control group, thus obtaining a CI threshold of 0.69. Still markedly higher frequencies of CI persist throughout the CHD spectrum. However, our data may help to interpret future studies on chronotropic index and CI using treadmill ergometer protocols in children and young adults.

As expected, in our study prevalence of CI was lowest in patients with minor lesions such as small shunt defects, or valvar defects, and was most present in patients with single ventricle circulation. Remarkably, the increase in prevalence of CI parallels the decline in peak oxygen consumption across the spectrum of CHD. Impaired heart rate response to exercise may therefore in part account for the diminished exercise capacity seen in these patients (22).

### Heart Rate Recovery

A deferred decrease of heart rate after cessation of exercise (heart rate recovery, HRR) is associated with increased mortality in adult patients with coronary artery disease and is a marker for poor outcome after pediatric heart transplantation (23). An increased risk of death with impaired HRR was even found in a subgroup of patients without heart failure or myocardial perfusion defects (24). Diller and colleagues revealed heart rate recovery as predictor of mortality also in an adult CHD cohort (6).

In this study we also detected lower HRR in children and adolescents with CHD and revealed an inverse correlation to peak oxygen consumption. Underlying mechanisms responsible for impaired HRR are not fully understood but may at least partly be attributed to an impaired autonomic function. Especially the immediate phase of HRR after cessation of exercise is thought to be promoted by vagal reactivation, which is followed by a sympathetic withdrawal during subsequent minutes (25).

This study highlights the relevance of heart rate response during exercise as an important piece of physiological response to extract from CPET alongside with conventional parameters. Whether specifically targeting abnormal heart rate reserve could improve exercise performance and affect prognosis remains unknown. Cardiac rehabilitation with structured training has been described to improve peak exercise capacity as well as early HRR in patients with repaired complex CHD (26). Preliminary data in pediatric pulmonary arterial hypertension patients suggest that exercise training may improve exercise performance at least in part by improved chronotropic competence (27). The impact of heart rate response targeting rehabilitation programs on longterm outcome needs to be evaluated in future studies.

### Study Limitations

The results of this study are limited by the lack of randomization and the relatively small number of patients. A referral bias where patients doing well have been preferentially selected cannot be ruled out with certainty, especially as we excluded patients on betablocker therapy and with pacemakers. However, in this study CPET was performed as part of routine evaluation in our pediatric cardiology outpatient clinic. Patients therefore were not restricted to any particular, narrow diagnostic group but rather the whole spectrum of CHD diagnoses was covered (including patients with mild, modest and complex lesions, as well as single ventricle and biventricular hemodynamics) and patients regardless of age, gender, history, and nature of surgery were included.

The formula for the chronotropic index by Wilkoff and colleagues might not be ideal for children and adolescents as it leads to an overestimation of chronotropic incompetence in young participants by using [220 − age] as formula to determine predicted maximum heart rate. Nevertheless, the formula by Wilkoff and Miller is to our knowledge the best concept for the assessment of chronotropic incompetence. To obtain a realistic cut-off for detection of chronotropic incompetence in childhood we adapted the threshold to the 2.5th percentile of our healthy population (in accordance with the methods used by Wilkoff and colleagues in their original publication). Clearly, the presented study is not adequately powered to obtain a new formula for the use in children and adolescents but our findings highlight the need for future prospective studies investigating heart rate response and chronotropic incompetence in this age group.

## Data Availability

All datasets generated for this study are included in the manuscript and/or the supplementary files.

## Ethics Statement

The study protocol was approved by the ethics committee of the University of Ulm, Helmholtzstr. 20, 89081 Ulm (reference number 77/18).

## Author Contributions

FvS: concept, data interpretation, drafting article, statistics, approval of the final version of this manuscript. SM: concept, data acquisition, statistics, critical revision, approval of the final version of this manuscript. JK: concept, data acquisition, critical revision, approval of the final version of this manuscript. AA, JS, PB, and MK: data acquisition, critical revision, approval of the final version of this manuscript. CA: concept, data interpretation, drafting article, approval of the final version of this manuscript.

### Conflict of Interest Statement

The authors declare that the research was conducted in the absence of any commercial or financial relationships that could be construed as a potential conflict of interest.
